# High-throughput analysis suggests differences in journal false discovery rate by subject area and impact factor but not open access status

**DOI:** 10.1186/s12859-020-03817-7

**Published:** 2020-12-09

**Authors:** L. M. Hall, A. E. Hendricks

**Affiliations:** 1 Mathematical and Statistical Sciences, University of Colorado Denver, Denver, CO USA; 2 Biostatistics and Informatics, Colorado School of Public Health, Aurora, CO USA

**Keywords:** False discovery rate, Replication, Reproducibility, Cancer, Open access, Impact factor

## Abstract

**Background:**

A low replication rate has been reported in some scientific areas motivating the creation of resource intensive collaborations to estimate the replication rate by repeating individual studies. The substantial resources required by these projects limits the number of studies that can be repeated and consequently the generalizability of the findings. We extend the use of a method from Jager and Leek to estimate the false discovery rate for 94 journals over a 5-year period using *p* values from over 30,000 abstracts enabling the study of how the false discovery rate varies by journal characteristics.

**Results:**

We find that the empirical false discovery rate is higher for cancer versus general medicine journals (*p* = 9.801E−07, 95% CI: 0.045, 0.097; adjusted mean false discovery rate cancer = 0.264 vs. general medicine = 0.194). We also find that false discovery rate is negatively associated with log journal impact factor. A two-fold decrease in journal impact factor is associated with an average increase of 0.020 in FDR (*p* = 2.545E−04). Conversely, we find no statistically significant evidence of a higher false discovery rate, on average, for Open Access versus closed access journals (*p* = 0.320, 95% CI − 0.015, 0.046, adjusted mean false discovery rate Open Access = 0.241 vs. closed access = 0.225).

**Conclusions:**

Our results identify areas of research that may need additional scrutiny and support to facilitate replicable science. Given our publicly available R code and data, others can complete a broad assessment of the empirical false discovery rate across other subject areas and characteristics of published research.

## Background

Increasing concern about the lack of reproducibility and replicability of published research [1–8] has led to numerous guidelines and recommendations including the formation of the National Academies of Sciences, Engineering, and Medicine committee [9] on Reproducibility and Replicability in Science [10–13]. In addition, efforts have been made to estimate the replication rate by forming large-scale collaborations to repeat a set of published studies within a particular discipline such as psychology [6], cancer biology [14], economics [15], and social sciences [16, 17]. The proportion of studies that replicate vary from approximately 1/3 to 2/3 depending, in part, on the power of the replication studies, the criteria used to define replication, and the proportion of true discoveries in the original set of studies [18].

These replication projects are often massive undertakings necessitating a large amount of resources and scientists. The sheer amount of resources needed can become a barrier limiting both the number and breadth of studies repeated. Indeed, the Cancer-Biology Reproducibility project lowered its projected number of studies for replication from 50 to 37 and then again to 18 [19]. This suggests that an efficient, complementary approach to evaluate replicability would be highly beneficial.

The false discovery rate, which is the number of scientific discoveries that are false out of all scientific discoveries reported, is a complementary measure to replicability since we expect a subset of true discoveries to replicate, but do not expect false discoveries to replicate. In 2013, Jager and Leek [20] published a method to estimate the empirical false discovery rate of individual journals using *p* values from abstracts. Compared to the resource intensive replication studies mentioned above, Jager and Leek’s method is quite efficient. Here, we take advantage of this efficiency to gather and use *p* values from over 30,000 abstracts to estimate the empirical false discovery rate for over 90 journals between 2011 and 2015. Using these journals, we evaluate if and how the empirical false discovery rate varies by three journal characteristics: (1) subject area—cancer versus general medicine; (2) 2-year journal impact factor (JIF), and (3) Open Access versus closed access.

1. *Subject Area*: The Cancer Biology Reproducibility Project was launched in October 2013 [14] after reports from several pharmaceutical companies indicated issues in replicating published findings in cancer biology. As indicated above, the Cancer Biology Replication Project has reduced the number of studies it plans to replicate by more than 50%. Here, we compare the empirical false discovery rate of cancer journals to general medicine journals, providing a complementary measure of the replication rate.
2. *Journal Impact Factor (JIF)*: Given limited resources, most projects that attempt to replicate published studies focus on high impact papers and journals in a handful of scientific fields. However, concerns about replicability occur throughout all fields of science and levels of impact. Indeed, research published in lower impact journals may have lower rates of replicability. Here, we evaluate if JIF is associated with the empirical false discovery rate of journals.
3. *Open Access versus closed access*: The prevalence of Open Access journals, where research is published and available to readers without a subscription or article fee, has increased considerably over the past decade [21]. The number of predatory journals, which exploit the Gold Open Access model by publishing with the primary purpose of collecting submission fees to make a profit, has also increased dramatically [22, 23]. While fees are common in Gold Open Access journals to remove pay walls, reputable Open Access journals have a thorough peer-review process while predatory journals have little to no peer review. Some have raised concerns that reputable Open Access journals may be letting peer-review standards fall to compete with predatory journals [23–27]. Here we evaluate whether Open Access journals from InCites [28] have a higher empirical false discovery rate than journals that are not Open Access (i.e. closed access).

## Results

The number of journals by subject area and Open Access status included in the final model is in Table 1. A full list of journals and descriptive information is included in Additional file 1: Table S6.

**Table 1:** Journal types

	Oncology	Medicine	Total
Open access	12	11	23
Closed access	45	26	71
Total	57	37	94

A nonlinear relationship, likely driven in part due to JIF being severely right skewed (Additional file 2: Fig. S3), was identified between JIF and the empirical false discovery rate. A natural logarithm transformation of JIF corrected the nonlinearity (Additional file 2: Fig. S5).

All 2-way and 3-way interactions were evaluated. More details are provided in the methods and supplemental. No interactions were nominally significant (*p* value > 0.05; Additional file 2: Appendix B). Therefore, the primary models only include main effect terms.

**Fig. 1:**
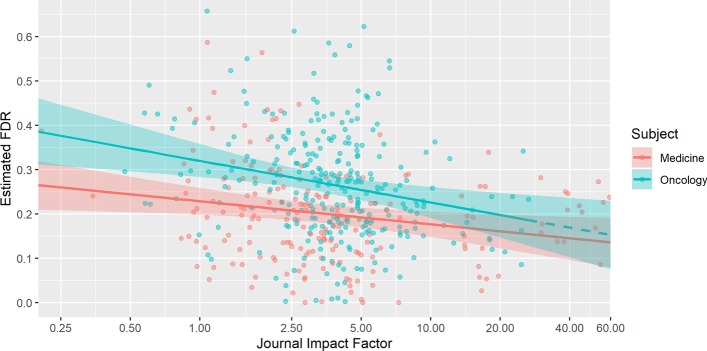
Relationship between JIF and false discovery rate by subject area. Estimated linear mixed effects regression from the stratified models with 95% bootstrapped confidence bands. General medicine journals (red), oncology journals (blue); solid line is the predicted relationship between false discovery rate and natural log of JIF adjusting for year and open access status. The dashed blue line represents extrapolated predictions beyond the observed maximum JIF of 26.51 for oncology journals

Results of the global model are shown in Table 2. Oncology journals have a significantly higher average empirical false discovery rate of 0.071 compared to general medicine journals. This equates to an adjusted mean false discovery rate of 0.264 versus 0.194 for cancer and general medicine journals respecitvely. In other words, cancer journals have a 36% times higher mean empirical false discovery rate compared to general medicine journals. Additionally, we find a significant inverse relationship between log JIF and estimated false discovery rate. The association is non-linear, with the effect being strongest at lower impact factors and diminishing at higher impact factors. For a twofold increase in JIF (e.g. JIF of 10 vs. 5), there is an average estimated decrease in empirical false discovery rate of 0.02. Figure 1 shows the relationship between JIF and empirical false discovery rate by journal subject area.

**Table 2:** Global model, all journal types

	Estimate	Std. Error	T-Value	*p* value	95% CI
Year	− 0.002	0.004	− 0.492	0.623	(− 0.009, 0.005)
Open Access	0.015	0.016	1.001	0.320	(− 0.015, 0.046)
Log(JIF)	− 0.029	0.008	− 3.797	2.545E−04	(− 0.044, − 0.014)
Oncology	0.071	0.013	5.257	9.801E−07	(0.045, 0.097)

We observe similar results in the oncology journal stratified model (Table 3) where we find a significant inverse association between estimated false discovery rate and log JIF. All else held constant, a two-fold decrease in oncology journal JIF is associated with an increase in false discovery rate of 0.028. A similar, although slightly weaker, relationship can be seen in the general medicine stratified model shown in Table 4, where a two-fold decrease in JIF is associated with an increase in false discovery rate of 0.016.

**Table 3:** Stratified: oncology journals

	Estimate	Std. Error	T-Value	*p* value	95% CI
Year	− 0.001	0.005	− 0.242	0.809	(− 0.011, 0.008)
Open Access	0.008	0.022	0.357	0.723	(− 0.035, 0.051)
Log(JIF)	− 0.041	0.013	− 3.136	0.003	(− 0.066, − 0.015)

**Table 4:** Stratified: medical journals

	Estimate	Std. Error	T-Value	*p* value	95% CI
Year	− 0.002	0.005	− 0.452	0.652	(− 0.013, 0.008)
Open Access	0.020	0.021	0.954	0.347	(− 0.021, 0.061)
Log(JIF)	-0.023	0.009	− 2.484	0.018	(− 0.040, − 0.005)

All secondary models show results and conclusions consistent with the models above and can be found in the supplemental materials (Additional file 2: Supplemental Materials and Tables S7–S24).

## Discussion

Using over 30,000 abstracts in 94 journals, we assessed whether journal subject area, impact factor, and Open Access status are associated with the empirical false discovery rate. We find significant and meaningful increase in the empirical false discovery rate for cancer versus general medicine journals and for journals with lower JIF. These results are in line with previous reports that suggest difficulty replicating published cancer research [2]. As these models assess the average relationship between journal characteristics and the empirical false discovery rate, these results do not implicate all oncology journals or journals with low JIF. Rather, these results suggest that more effort and higher standards are needed in the field of oncology research and that special attention may be needed for journals with lower impact factors.

We find no statistically significant evidence of a relationship between Open Access status and false discovery rate. This result does not preclude the possibility that a small number of Open Access journals have a high false discovery rate. Rather this result suggests that, after adjusting for JIF and journal subject area, there is no significant evidence of a systematically higher empirical false discovery rate across all Open Access journals evaluated here or of a small number of Open Access journals with extremely high empirical false discovery rates.

There are several limitations to our study. We do not investigate patterns in the estimated false discovery rates for individual journals; rather, we assess whether certain journal characteristics (i.e. subject area, journal impact factor, Open Access status) are associated, on average, with empirical false discovery rate. Additionally, this study was performed on a sample of English-speaking journals from the field of medical research with Open Access journals from InCites for each subject area of interest. While outside of the scope of this study, increasing the sample to include non-English speaking journals, other subject areas within medicine, or repeating the study in subject areas outside of medicine would provide additional information about the relationship between the empirical false discovery rate and journal characteristics. Finally, while our inclusion of Open Access status was motivated by the increase in predatory journals, we do not directly study predatory journals here. We anticipate that our sample may underrepresent predatory journals as predatory journals are often excluded from reputable journal curation sites such as InCites. Further, restricting to English-speaking journals may exclude the majority of predatory journals that have been shown to originate in Asia and Africa [29, 30].

As Leek and Jager state in their 2017 Annual Review Stats paper [31], *p* values can be presented and even manipulated in ways that can influence or call into question the accuracy of their method’s false discovery rate estimates. Here, we do not focus on the accuracy and precision of individual *p* values and false discovery rates. Instead, we compare the average false discovery rate estimates by various journal characteristics. A critical assumption for our models is that any bias in the *p* values is consistent between journals. It is possible, although we believe unlikely, that journal characteristics not related to the false discovery rate may change the distribution of observed *p* values and thus influence the estimated false discovery rate.

We were able to complete the research presented here because Jager and Leek adhered to the highest standards of reproducible research by making their code publicly available and providing complete statistical methods. We strive to do the same here by providing complete statistical details in the supplemental section and our R code on GitHub (https://github.com/laurenhall/fdr). We hope that others will use our code and statistical details to improve upon our work and to complete research investigating patterns in the empirical false discovery rate.

## Conclusions

Here, we investigated the relationship between the empirical false discovery rate of journals and journal subject area, JIF, and Open Access status. We find that cancer journals have a higher empirical false discovery rate compared to general medicine journals and that the empirical false discovery rate is inversely related to JIF. We do not find significant evidence of different empirical false discovery rates for Open Access versus closed access journals. Given its efficiency and ability to incorporate a large and comprehensive set of published studies, the statistical framework we use here is complementary to large-scale replication studies. We hope that our approach will enable other researchers to assess the empirical false discovery rate across a wider array of disciplines and journal attributes. We believe this will provide insights into the patterns of replicability across science and ultimately guidance as to where more resources, higher standards, and training are needed.

## Methods

### Methodological framework

Jager and Leek [20] define false discovery rate as the proportion of results reported as significant where the null hypothesis is actually true (i.e. the result is a false discovery). This definition is similar to the traditional definition of false discovery rate by Benjamini and Hochberg in 1995 [32] where Q, the proportion of errors due to rejecting a true null hypothesis, is estimated as the expected number of true null hypotheses divided by the total number of significant tests. While the false discovery rate definitions are similar, the estimation methods differ. Jager and Leek exploit the close relationship between false discovery rate and empirical Bayes methods as described by others including Efron and Tibshirani [33]. More details about Jager and Leek’s method to estimate false discovery rate can be found in their original publication [20].

Jager and Leek’s method uses *p* values from abstracts to arrive at an empirical false discovery rate estimate per journal per year. *P* values that fall below a given significance threshold, α, are defined as positive test results and are included in the false discovery rate estimation. Within this set of positive test results, results can be true or false. True discovery *p* values are assumed to follow a truncated Beta distribution (tBeta) with possible observable values between 0 and *α* and with shape parameters *a* and *b*. False discoveries are assumed to follow a uniform distribution (U) between 0 and α. The true discovery and false discovery distributions are combined with mixing parameter π_0_, which is the proportion of *p* values that belong to the Uniform (false discovery) distribution. If we assume that the distribution of *p* values is continuous on the interval (0, 1), the combined distribution for all positive test results (i.e. *p* values less than α) is:

$${\text{f}}({\text{p}}|{\text{a}},{\text{b}},\pi_{0} ) = \pi_{0} U\left( {0,\alpha } \right) + \left( {1 - \pi_{0} } \right){\text{tBeta}}\left( {{\text{a}},{\text{b}}} \right)$$

where *a* > 0*, b* > 0 and 0 < π_0_ < 1. Using the Expectation–Maximization (EM) algorithm, the maximum likelihood estimates are simultaneously estimated for the shape parameters *a, b* and the false discovery rate, π_0_. Journal articles often do not report exact *p* values (e.g. *p* = 0.0123); adjustments are made to the likelihood function to accommodate rounded (e.g. *p* = 0.01) or truncated *p* values (e.g. *p* < 0.05). Two indicator variables are used to indicate either rounded *p* values or truncated *p* values. *P* values that are rounded or truncated have their likelihood evaluated by integrating over all values that could possibly lead to the reported value (e.g., for *p* < 0.05, the associated probability is $\mathop \smallint \limits_{0}^{0.05} f\left( {p|a,b,\pi_{0} } \right)dp$; for *p* = 0.01, the associated probability is $\mathop \smallint \limits_{0.005}^{0.015} f\left( {p|a,b,\pi_{o} } \right)dp$). *P* values are classified as rounded if the reported value has two or fewer decimal places, and as truncated if the value was read following a < or ≤ character in the text. For more details, see the Supplemental Materials of Jager and Leek [20].

### Application

**Fig. 2:**
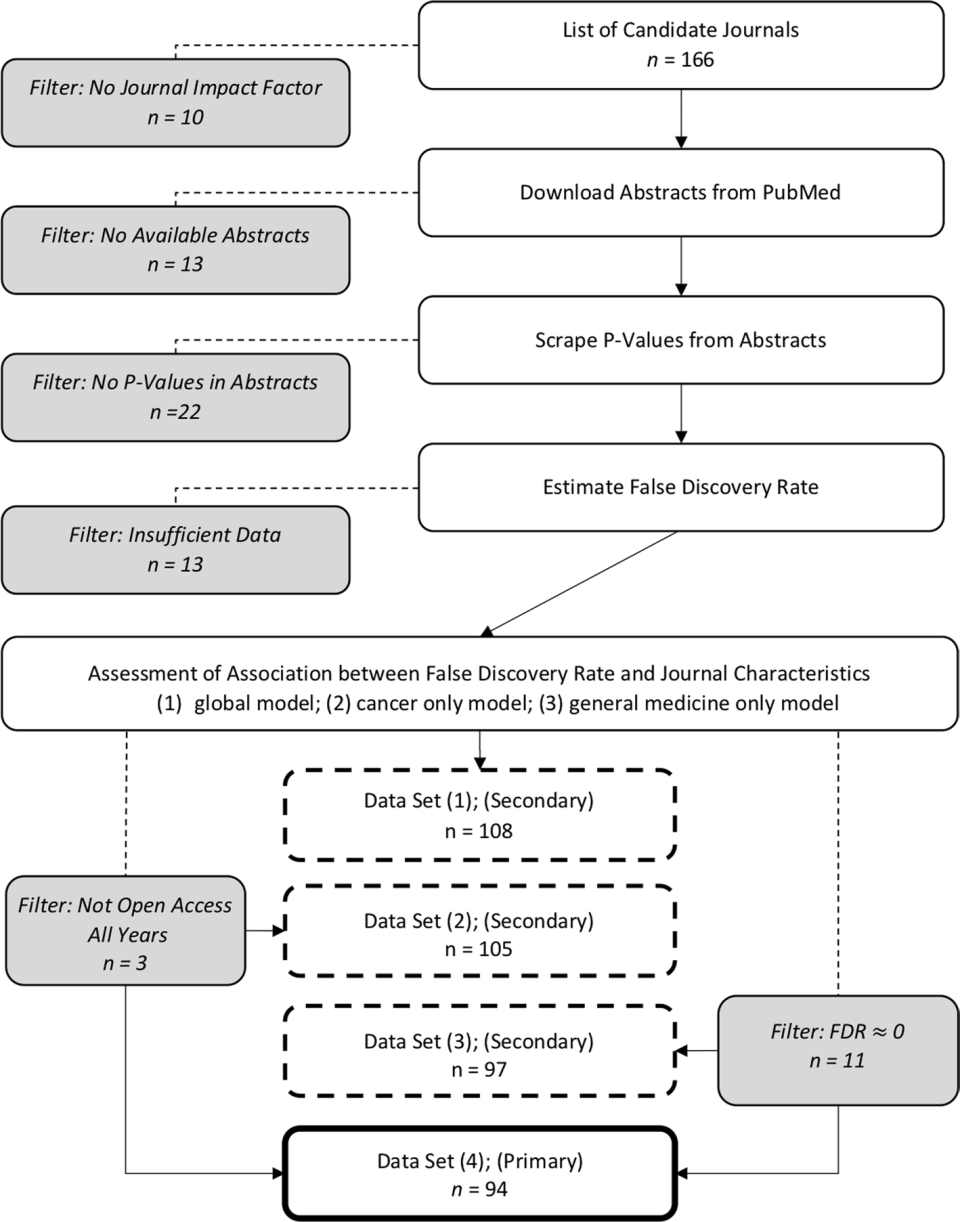
Flowchart for filtering and analysis. Gray boxes represent filtering steps. One primary dataset (bold, solid outline) and three secondary datasets (bold, dashed outline) were used to assess association between journal empirical false discovery rate and journal characteristics

We selected journals from InCites [28] using the following criteria for each journal during the years 2011–2015: available 2-year JIF score, published in English, categorized as General & Internal medicine, Research & Experimental medicine, or Oncology according to InCite’s subject tags, and listed as available on the PubMed online database as of August 2017. False discovery rate was calculated on 143 journals. The EM algorithm did not converge for one or more years for 35 journals, resulting in no false discovery rate estimate. These journals were removed from further consideration, resulting in a final sample of 108 journals with 36,565 abstracts. InCites was used to classify journals as Open Access or closed access for each year of the study. For example, a journal marked as “Open Access since 2013” will be marked as Open Access only for the years 2013–2015. All available abstracts from 2011–2015 were collected from the online PubMed database using E-Utilities from the National Center for Biotechnology Information [34]. A flowchart visualizing our filtering and analysis process is in Fig. 2. For more details on journal selection, see Additional file 2: Supplemental Materials and Additional file 3: Table S1.

Similar to Jager and Leek, *p* values were scraped from the abstracts using regular expressions, which searched the abstract text for incidences of the non-case-sensitive phrases “p = ”, “p < ”, and “p ≤ ”. The strings following these phrases were collected and presumed to be a reported *p* value. These strings were cleaned to remove excess punctuation or spacing characters and the remaining value was converted to numeric entry in scientific notation. The source code provided by Jager and Leek [20] was updated to include additional standardizing of notation and formatting in the abstracts, including scientific notation, punctuation, and spacing characters, before searching for *p* values. This reduced the number of search errors from misread characters. Other than this addition, no changes were made to Jager and Leek’s original algorithm for estimating false discovery rate. Details, including all notational substitutions, can be found in the source code available at https://github.com/laurenhall/fdr.

To identify and estimate differences in false discovery rate by journal characteristic, we applied linear mixed effects models with the estimated false discovery rate as the outcome and a random intercept by journal to account for multiple observations from each journal for each year as shown in Eq. (1).

1 $${\varvec{Y}} = \user2{X\beta } + b + {\varvec{\epsilon}}$$

where ${\varvec{Y}}$ is a vector of the empirical false discovery rate for N abstracts; ${\varvec{X}}$ is a $N \times p$ matrix of p predictors including two-way and three-way interactions where applicable; $b$ is a random intercept for journal.

We fit three models: one global model with journal subject area as a covariate (1 for oncology and 0 otherwise), and two models stratified by journal subject area (oncology and medicine). Within each model, the following covariates were included: year, JIF, and Open Access status (1 if Open Access and 0 otherwise). To correct for a non-linear relationship between JIF and empirical false discovery rate, we used a natural log transformation of JIF. Additionally, two-way and three-way interaction terms between journal subject area, Open Access status, and JIF were considered for the global model, and a two-way interaction between Open Access status and JIF was considered for each stratified model. We first assessed the three-way interaction and then each nested two-way interaction removing any that did not contribute significantly to the model (i.e. *p* value ≤ 0.05). All main effects were left in the model regardless of significance. The final model was compared with the intermediate models to assess consistency of results. Details for the intermediate models including results are in Additional file 2: Appendix B in Supplemental Materials). A nominal significance threshold of α = 0.05 was used to assess significance.

To check for consistency and to ensure that our results were not driven by unusual journal characteristics, each of the three models was fit to four data sets: (1) all journals (N = 108); (2) excluding journals that were not Open Access for all five study years (N = 105); (3) excluding journals that produced an estimated false discovery rate of approximately zero (N = 97); (4) excluding both Open Access journals that were not Open Access for all five study years and journals that produced an estimated false discovery rate of approximately zero (N = 94). Models using data from (4) are shown in the Results section. More details and results for datasets (1), (2), and (3) are in Additional file 2: Appendix A. Descriptive statistics, and distributions of these four datasets are in Additional file 2: Tables S2–S5 and Additional file 2: Figs. S1–S4.

## Supplementary information

**Additional file 1**. Supplemental Table S6: a full list of all considered journals organized by subject, open access group membership status, and whether the journal was removed.

**Additional file 2**. Contains detailed journal selection criteria including Supplemental Tables S2–S5, model selection and models for additional data sets including Supplemental Tables S7–S24, and Supplemental Figures S1–S5.

**Additional file 3**. Supplemental Table S1: The full list of journals by year, including estimated false discovery rate, number of abstracts received, and number of p-values identified in abstracts.

## Data Availability

Data, code, and details are available at https://github.com/laurenhall/fdr. Detailed journal selection criteria is in Additional file 2: Appendix A.

## Abbreviation

JIF: Journal impact factor
