# Supplementary material for: High-throughput analysis suggests differences in journal false discovery rate by subject area and impact factor but not open access status
Source: BMC Bioinformatics. 2020 Dec 9;21:564. doi: 10.1186/s12859-020-03817-7 (PMC7724881; doi:10.1186/s12859-020-03817-7)
Supplement: Supplementary file 2 — Additional file 2. Contains detailed journal selection criteria including Supplemental Tables S2–S5, model selection and models for additional data sets including Supplemental Tables S7–S24, and Supplemental Figures S1–S5. [file 12859_2020_3817_MOESM2_ESM.docx]

**Supplementary Materials for High-throughput application of method to estimate and compare false discovery rates in journals**

**Appendix A: Detailed journal selection criteria**

One hundred and sixty-six journals were identified by accessing the Thompson-Reuters InCites journal list of journals that had a subject marked as “Oncology”, “Medicine – General & Internal”, or “Medicine – Research & Experimental”. The full list of journals is in the supplementary file “Journals.csv” (<https://github.com/laurenhall/fdr>) and was compiled on August 23, 2017.

The abstracts for these journals were scraped from PubMed for the years 2011-2015. Journals that had no abstracts available for one of more of these years were eliminated (13 journals): *Advancement in Clinical and Experimental Medicine, Analytic Cell Pathology, Canadian Medical Association Journal, Cancer Immunology Research, Cancer Prevention Research, Cellular Oncology, Clinical Medicine, Concoimmunology, Experimental Biological Medicine, Gynaecological Oncology, Journal of Diabetes Research, Molecular Therapy – Nucleic Acids,* and *Sexual Medicine*.

We estimated the false discovery rate for the remaining 153 journals for each of the five research years. Some journals, due to either a small number of p-values or not enough unique observations (as can occur when most reported p-values are truncated), did not produce false discovery estimates for one or more years. These journals were eliminated (35 journals): *Cancer Cell, Cancer Cell International, Cancer Discovery, Cancer Imaging, Cancer Journal, Clinical Journal of Oncological Nursing, Current Molecular Medicine, Discovery Medicine, EMBO Molecular Medicine, European Journal of General Practice, Experimental Cell Research, Family Medicine, Future Oncology, Journal of Cancer Education, Journal of Clinical Investigation, Journal of Experimental Medicine, Journal of Genetic Medicine, Journal of Immunotherapy, Journal of International Medical Research, Journal of the National Comprehensive Cancer Network, Journal of the National Medical Association, Journal of the Royal Society of Medicine, MAbs, Molecular Aspects in Medicine, Nature Medicine, Oncology Research, Pigment Cell Melanoma Research, Primary Care, Seminars in Cancer Biology, Seminars in Oncology, Seminars in Radiation and Oncology, Statistics in Medicine, Stem Cell Research and Therapy, Theranostics,* and *Trends in Molecular Medicine.*

In addition to the above journals, several journals were removed from consideration due to not having a two-year journal impact factor (JIF) score available for one or more of the study years (10 journals): *American Journal of Cancer Research, Blood Cancer Journal, BMJ Open, Cancer Research and Treatment, Clinical Epigenetics, Clinics, Hormones and Cancer, International Journal of Clinical and Experimental Medicine, Journal of Cancer,* and *Journal of Ophthalmology.*

The remaining 108 journals were included in the study. The full list of journals by year, including estimated false discovery rate, number of abstracts received, and number of p-values identified in abstracts, can be found in **Table S1**. **Table S2** below describes the distribution of journals present in this data set by subject and Open Access status. Journals which were Open Access in some years and not others count towards both categories, proportional to how many years they spent in each state.

**Table S2** – Number of Journals by Subject and Open Access status

|  | Oncology | Medicine | Total |
| --- | --- | --- | --- |
| Open Access | 12 | 11 | 23 |
| Closed Access | 49 | 30 | 79 |
| Both | 2 | 4 | 6 |
| Total | 63 | 45 | 108 |

**Reduced data sets**

In addition to the full data set consisting of 108 journals described in the previous section (i.e. dataset 1), three reduced data sets were used in this study. These data sets have one or both of the following groups of journals removed:

2) Remove journals with FDR estimates close to zero: As stated in the methods, the EM-algorithm is estimating the proportion of false discovery p-values is limited to between 0 and 1 and cannot equal 0 or 1. Estimates of the number of false discovery p-values close to zero may instead be zero. An estimated false discovery rate close to zero implies that no p-values in the sample were false discoveries. We removed journals if the expected number of false discovery p-values for a journal in a year was less than 0.01. The expected number of false discovery p-values was calculated by multiplying the total number of p-values times the estimated FDR.

Journals excluded from this data set (**Table S1**): *Br J Gen Pract Medicine, Clin Trials Medicine, Curr Oncol Oncology, Eur J Med Res Medicine, J Cell Mol Med Medicine, Mol Cancer Res Oncology, Mol Oncol Oncology, Mol Ther Medicine, Nanomedicine Medicine, Surg Oncol Oncology, Thorac Cancer Oncology*

**Table S3** below shows the distribution of journals present in this data set by subject and Open Access status.

**Table S3** – Number of Journals by Subject and Open Access status after removing journals with FDR close to zero

|  | Oncology | Medicine | Total |
| --- | --- | --- | --- |
| Open Access | 12 | 11 | 23 |
| Closed Access | 45 | 26 | 71 |
| Both | 1 | 2 | 3 |
| Total | 58 | 39 | 97 |

3) Remove journals that were not consistently Open Access or closed access during the study years: Some journals switched to an Open Access model during the study years. In the unfiltered data set, these journals are encoded with a 1 for Open Access for the years they were Open Access, and 0 otherwise. For this data subset, we removed these journals.

Journals excluded from this data set (**Table S1**): *Cancer Sci Oncology, Eur J Med Res Medicine, J Cell Mol Med Medicine, J Res Med Sci Medicine, Medicine, Thorac Cancer Oncology*.

**Table S4** below describes the distribution of journals present in this data set by subject and Open Access status.

**Table S4** – Number of Journals by Subject and Open Access status after removing journals that switched Open Access status

|  | Oncology | Medicine | Total |
| --- | --- | --- | --- |
| Open Access | 12 | 11 | 23 |
| Closed Access | 49 | 30 | 79 |
| Total | 61 | 41 | 102 |

4) Reduced data set excluding both journals from 2 and 3: This data set excludes both sets of journals described above and is the primary dataset presented in the paper. **Table S5** below describes the distribution of journals present in this data set by subject and Open Access status.

**Table S5** – Number of Journals by Subject and Open Access status after removing journals that switched Open Access status and had FDR estimates close to zero

|  | Oncology | Medicine | Total |
| --- | --- | --- | --- |
| Open Access | 12 | 11 | 23 |
| Closed Access | 45 | 26 | 71 |
| Total | 57 | 37 | 94 |

Additionally, a full list of all considered journals organized by subject, open access group membership status, and whether the journal was removed can be found in **Table S6**.

**Appendix B: Model Selection and Models for Additional Data Sets**

As described in the main text, we arrived at our final model through a modified selection method. Beginning with a full model including all two-way and three-way (where appropriate) interaction terms, we removed any interactions that were not significant beginning with the three-way terms and then the two-way terms, beginning with the least significant. No main effects were removed from any of the models. The tables below contain the stages of model selection not shown in the main text, as well as models for the data sets described in the previous section to demonstrate consistency of results. In the models that follow, a natural logarithm transformation is applied to JIF as the transformation was found to improve the linearity of the relationship between JIF and estimated FDR (**Figure S5**).

**Global Model**

**Model Selection Details – Main Text Models**

**Table S7** – Full Model, Journals with Zero Estimates and Inconsistent Open Access Status Removed

|  | **Estimate** | **Std. Error** | **T-Value** | **P-Value** |
| --- | --- | --- | --- | --- |
| **Intercept** | 3.891 | 7.309 | 0.532 | 0.595 |
| **Year** | -0.002 | 0.004 | -0.503 | 0.615 |
| **Open Access** | 0.059 | 0.038 | 1.543 | 0.126 |
| **Log(JIF)** | -0.018 | 0.011 | -1.665 | 0.099 |
| **Oncology** | 0.114 | 0.029 | 3.983 | 1.367E-04 |
| **OA * Log(JIF)** | -0.034 | 0.027 | -1.257 | 0.212 |
| **OA * Onc.** | -0.094 | 0.056 | -1.688 | 0.095 |
| **Log(JIF) * Onc.** | -0.030 | 0.017 | -1.754 | 0.083 |
| **OA * Log(JIF) * Onc.** | 0.074 | 0.042 | 1.761 | 0.081 |

**Table S8** – Journals with Zero Estimates and Inconsistent Open Access Status Removed, Excluding 3-Way Interaction

|  | **Estimate** | **Std. Error** | **T-Value** | **P-Value** |
| --- | --- | --- | --- | --- |
| **Intercept** | 3.591 | 7.309 | 0.491 | 0.624 |
| **Year** | -0.002 | 0.004 | -0.461 | 0.645 |
| **Open Access** | 0.024 | 0.033 | 0.730 | 0.467 |
| **Log(JIF)** | -0.022 | 0.010 | -2.154 | 0.034 |
| **Oncology** | 0.097 | 0.027 | 3.547 | 6.146E-04 |
| **OA * Log(JIF)** | -0.003 | 0.021 | -0.159 | 0.874 |
| **OA * Onc.** | -0.012 | 0.031 | -0.398 | 0.692 |
| **Log(JIF) * Onc.** | -0.018 | 0.016 | -1.106 | 0.272 |

**Table S9** – Journals with Zero Estimates and Inconsistent Open Access Status Removed, Excluding OA * Log(JIF) Interaction

|  | **Estimate** | **Std. Error** | **T-Value** | **P-Value** |
| --- | --- | --- | --- | --- |
| **Intercept** | 3.585 | 7.309 | 0.490 | 0.624 |
| **Year** | -0.002 | 0.004 | -0.461 | 0.645 |
| **Open Access** | 0.020 | 0.023 | 0.892 | 0.375 |
| **Log(JIF)** | -0.023 | 0.010 | -2.321 | 0.022 |
| **Oncology** | 0.097 | 0.027 | 3.587 | 5.353E-04 |
| **OA * Onc.** | -0.012 | 0.031 | -0.396 | 0.693 |
| **Log(JIF) * Onc.** | -0.018 | 0.016 | -1.129 | 0.262 |

**Table S10** –Journals with Zero Estimates and Inconsistent Open Access Status Removed, Excluding OA*SUBJ Interaction

|  | **Estimate** | **Std. Error** | **T-Value** | **P-Value** |
| --- | --- | --- | --- | --- |
| **Intercept** | 3.608 | 7.308 | 0.494 | 0.622 |
| **Year** | -0.002 | 0.004 | -0.464 | 0.643 |
| **Open Access** | 0.014 | 0.015 | 0.886 | 0.378 |
| **Log(JIF)** | -0.023 | 0.010 | -2.367 | 0.020 |
| **Oncology** | 0.092 | 0.024 | 3.811 | 2.370E-04 |
| **Log(JIF) * Onc.** | -0.017 | 0.016 | -1.083 | 0.282 |

**Table S11** – Final Model, Journals with Zero Estimates and Inconsistent Open Access Status Removed, Excluding Log(JIF) * Subject Interaction

|  | **Estimate** | **Std. Error** | **T-Value** | **P-Value** | **95% CI** |
| --- | --- | --- | --- | --- | --- |
| **Intercept** | 3.816 | 7.292 | 0.523 | 0.601 | (-10.496, 18.119) |
| **Year** | -0.002 | 0.004 | -0.492 | 0.623 | (-0.009, 0.005) |
| **Open Access** | 0.015 | 0.016 | 1.001 | 0.320 | (-0.015, 0.046) |
| **Log(JIF)** | -0.029 | 0.008 | -3.797 | 2.545E-04 | (-0.044, -0.014) |
| **Oncology** | 0.071 | 0.013 | 5.257 | 9.801E-07 | (0.045, 0.097) |

**Final Combined Models – Secondary Data Sets**

Data Set 1: All Journals

**Table S12** – Final Model, All Journals

|  | **Estimate** | **Std. Error** | **T-Value** | **P-Value** | **95% CI** |
| --- | --- | --- | --- | --- | --- |
| **Intercept** | -6.215 | 7.226 | -0.860 | 0.390 | (-20.392, 0.050) |
| **Year** | 0.003 | 0.004 | 0.891 | 0.374 | (-0.004, 0.010) |
| **Open Access** | 0.019 | 0.015 | 1.312 | 0.192 | (-0.009, 0.047) |
| **Log(JIF)** | -0.030 | 0.007 | -4.068 | 8.785E-05 | (-0.044, -0.016) |
| **Oncology** | 0.079 | 0.013 | 6.237 | 9.654E-09 | (0.054, 0.103) |

Data Set 2: Journals with Zero Estimates removed

**Table S13** – Final Model, Zero Estimates Removed

|  | **Estimate** | **Std. Error** | **T-Value** | **P-Value** | **95% CI** |
| --- | --- | --- | --- | --- | --- |
| **Intercept** | 2.229 | 7.147 | 0.312 | 0.755 | (-11.799, 16.243) |
| **Year** | -9.932E-04 | 0.004 | -0.280 | 0.780 | (-0.008, 0.006) |
| **Open Access** | 0.019 | 0.015 | 1.258 | 0.211 | (-0.010, 0.048) |
| **Log(JIF)** | -0.031 | -0.008 | -4.020 | 1.116E-04 | (-0.045, -0.016) |
| **Oncology** | 0.072 | 0.013 | 5.441 | 4.258E-07 | (0.047, 0.098) |

Data Set 3: Journals with Inconsistent Open Access Status removed

**Table S14** – Final Model, Inconsistent Open Access Status Removed

|  | **Estimate** | **Std. Error** | **T-Value** | **P-Value** | **95% CI** |
| --- | --- | --- | --- | --- | --- |
| **Intercept** | -4.155 | 7.349 | -0.565 | 0.572 | (-18.576, 10.260) |
| **Year** | 0.002 | 0.004 | 0.595 | 0.552 | (-0.005, 0.009) |
| **Open Access** | 0.019 | 0.016 | 1.231 | 0.221 | (-0.011, 0.049) |
| **Log(JIF)** | -0.029 | 0.008 | -3.669 | 3.823E-04 | (-0.044, -0.013) |
| **Oncology** | 0.077 | 0.013 | 5.874 | 5.849E-08 | (0.051, 0.102) |

**Stratified Models – Oncology**

**Model Selection Details – Main Text Models**

**Table S15** – Oncology Full Model, Journals with Zero Estimates and Inconsistent Open Access Status Removed

|  | **Estimate** | **Std. Error** | **T-Value** | **P-Value** |
| --- | --- | --- | --- | --- |
| **Intercept** | 2.955 | 9.875 | 0.299 | 0.765 |
| **Year** | -0.001 | 0.005 | -0.266 | 0.790 |
| **Open Access** | -0.035 | 0.042 | -0.843 | 0.403 |
| **Log(JIF)** | -0.048 | 0.014 | -3.386 | 0.001 |
| **OA * Log(JIF)** | 0.040 | 0.033 | 1.205 | 0.233 |

**Table S16** – Oncology Final Model, Journals with Zero Estimates and Inconsistent Open Access Status Removed, excluding OA * JIF interaction

|  | **Estimate** | **Std. Error** | **T-Value** | **P-Value** | **95% CI** |
| --- | --- | --- | --- | --- | --- |
| **Intercept** | 2.704 | 9.862 | 0.274 | 0.784 | (-16.691, 22.041) |
| **Year** | -0.001 | 0.005 | -0.242 | 0.809 | (-0.011, 0.008) |
| **Open Access** | 0.008 | 0.022 | 0.357 | 0.723 | (-0.035, 0.051) |
| **Log(JIF)** | -0.041 | 0.013 | -3.136 | 0.003 | (-0.066, -0.015) |

**Final Stratified Models – Oncology – Secondary Data Sets**

Data Set 1: All Journals

**Table S17** – Oncology Final Model, All Journals

|  | **Estimate** | **Std. Error** | **T-Value** | **P-Value** | **95% CI** |
| --- | --- | --- | --- | --- | --- |
| **Intercept** | -8.336 | 10.032 | -0.831 | 0.407 | (-28.059, 11.329) |
| **Year** | 0.004 | 0.005 | 0.862 | 0.390 | (-0.005, 0.014) |
| **Open Access** | 0.015 | 0.021 | 0.713 | 0.478 | (-0.026, 0.057) |
| **Log(JIF)** | -0.035 | 0.012 | -2.860 | 0.006 | (-0.060, -0.011) |

Data Set 2: Journals with Zero Estimates removed

**Table S18** – Oncology Final Model, Zero Estimates Removed

|  | **Estimate** | **Std. Error** | **T-Value** | **P-Value** | **95% CI** |
| --- | --- | --- | --- | --- | --- |
| **Intercept** | 1.790 | 9.770 | 0.183 | 0.855 | (-17.424, 20.939) |
| **Year** | -7.317E-04 | 0.005 | -0.151 | 0.880 | (-0.010, 0.009) |
| **Open Access** | 0.011 | 0.022 | 0.519 | 0.605 | (-0.031, 0.054) |
| **Log(JIF)** | -0.040 | 0.013 | -3.021 | 0.004 | (-0.066, -0.014) |

Data Set 3: Journals with Inconsistent Open Access Status removed

**Table S19** – Oncology Final Model, Inconsistent Open Access Status Removed

|  | **Estimate** | **Std. Error** | **T-Value** | **P-Value** | **95% CI** |
| --- | --- | --- | --- | --- | --- |
| **Intercept** | -5.130 | 10.126 | -0.507 | 0.613 | (-25.034, 14.729) |
| **Year** | 0.003 | 0.005 | 0.537 | 0.592 | (-0.007, 0.013) |
| **Open Access** | 0.009 | 0.022 | 0.422 | 0.675 | (-0.033, 0.051) |
| **Log(JIF)** | -0.037 | 0.013 | -2.919 | 0.005 | (-0.062, -0.012) |

**Stratified Models – Medicine**

**Model Selection Details – Main Text Models**

**Table S20** – Medicine Full Model, Journals with Zero Estimates and Inconsistent Open Access Status Removed

|  | **Estimate** | **Std. Error** | **T-Value** | **P-Value** |
| --- | --- | --- | --- | --- |
| **Intercept** | 5.483 | 10.686 | 0.513 | 0.609 |
| **Year** | -0.003 | 0.005 | -0.493 | 0.623 |
| **Open Access** | 0.060 | 0.036 | 1.657 | 0.107 |
| **Log(JIF)** | -0.017 | 0.010 | -1.765 | 0.086 |
| **OA * Log(JIF)** | -0.035 | 0.026 | -1.358 | 0.183 |

**Table S21** – Medicine Final Model, Journals with Zero Estimates and Inconsistent Open Access Status Removed, excluding OA * JIF interaction

|  | **Estimate** | **Std. Error** | **T-Value** | **P-Value** | **95% CI** |
| --- | --- | --- | --- | --- | --- |
| **Intercept** | 5.068 | 10.718 | 0.473 | 0.637 | (-15.986, 26.151) |
| **Year** | -0.002 | 0.005 | -0.452 | 0.652 | (-0.013, 0.008) |
| **Open Access** | 0.020 | 0.021 | 0.954 | 0.347 | (-0.021, 0.061) |
| **Log(JIF)** | -0.023 | 0.009 | -2.484 | 0.018 | (-0.040, -0.005) |
|  |  |  |  |  |  |

**Final Stratified Models – Medicine – Secondary Data Sets**

Data Set 1: All Journals

**Table S22** – Medicine Final Model, All Journals

|  | **Estimate** | **Std. Error** | **T-Value** | **P-Value** | **95% CI** |
| --- | --- | --- | --- | --- | --- |
| **Intercept** | -3.394 | 10.247 | -0.331 | 0.741 | (-23.490, 16.753) |
| **Year** | 0.002 | 0.005 | 0.353 | 0.725 | (-0.008, 0.012) |
| **Open Access** | 0.021 | 0.020 | 1.083 | 0.284 | (-0.017, 0.060) |
| **Log(JIF)** | -0.027 | 0.009 | -3.038 | 0.004 | (-0.045, -0.010) |

Data Set 2: Journals with Zero Estimates removed

**Table S23** – Medicine Final Model, Zero Estimates Removed

|  | **Estimate** | **Std. Error** | **T-Value** | **P-Value** | **95% CI** |
| --- | --- | --- | --- | --- | --- |
| **Intercept** | 2.518 | 10.346 | 0.243 | 0.808 | (-17.809, 22.844) |
| **Year** | -0.001 | 0.005 | -0.222 | 0.825 | (-0.011, 0.009) |
| **Open Access** | 0.024 | 0.020 | 1.196 | 0.238 | (-0.015, 0.063) |
| **Log(JIF)** | -0.025 | 0.009 | -2.904 | 0.006 | (-0.042, -0.009) |

Data Set 3: Journals with Inconsistent Open Access Status removed

**Table S24** – Medicine Final Model, Inconsistent Open Access Status Removed

|  | **Estimate** | **Std. Error** | **T-Value** | **P-Value** | **95% CI** |
| --- | --- | --- | --- | --- | --- |
| **Intercept** | -3.054 | 10.448 | -0.292 | 0.770 | (-23.570, 17.492) |
| **Year** | 0.002 | 0.005 | 0.313 | 0.755 | (-0.009, 0.012) |
| **Open Access** | 0.028 | 0.022 | 1.234 | 0.225 | (-0.016, 0.071) |
| **Log(JIF)** | -0.024 | 0.010 | -2.506 | 0.016 | (-0.043, -0.006) |

**Appendix C: Supplemental Figures**

**Distribution of Scraped P-Values**


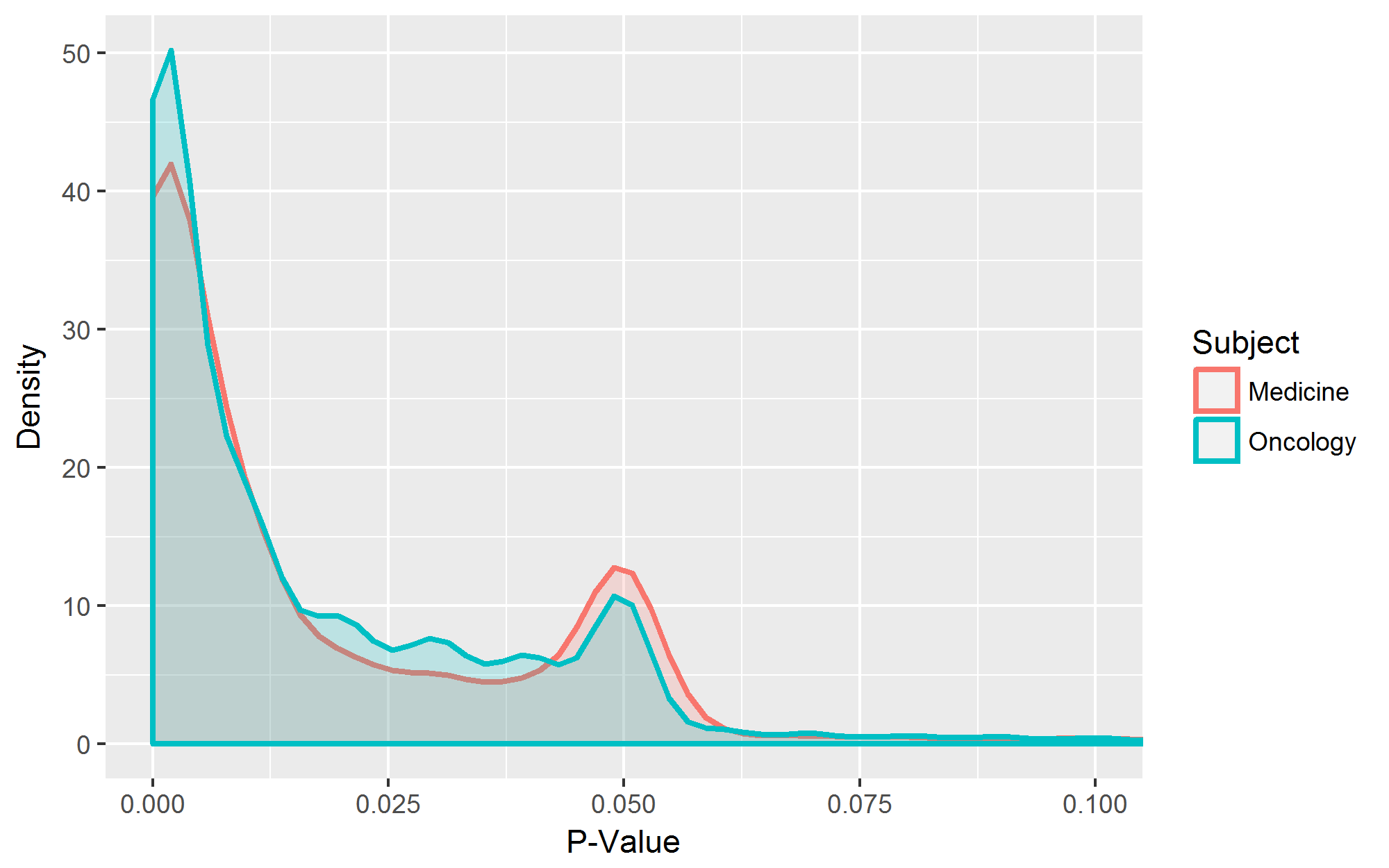


**Figure S1 – Distribution of scraped p-values between 0 and 0.1 by Subject.** All journals and all years included. Medicine journals (red) and Oncology journals (blue). The peaks at 0.001 and 0.05 represent large numbers of p-values reported as P < 0.001 or P < 0.05, respectively.


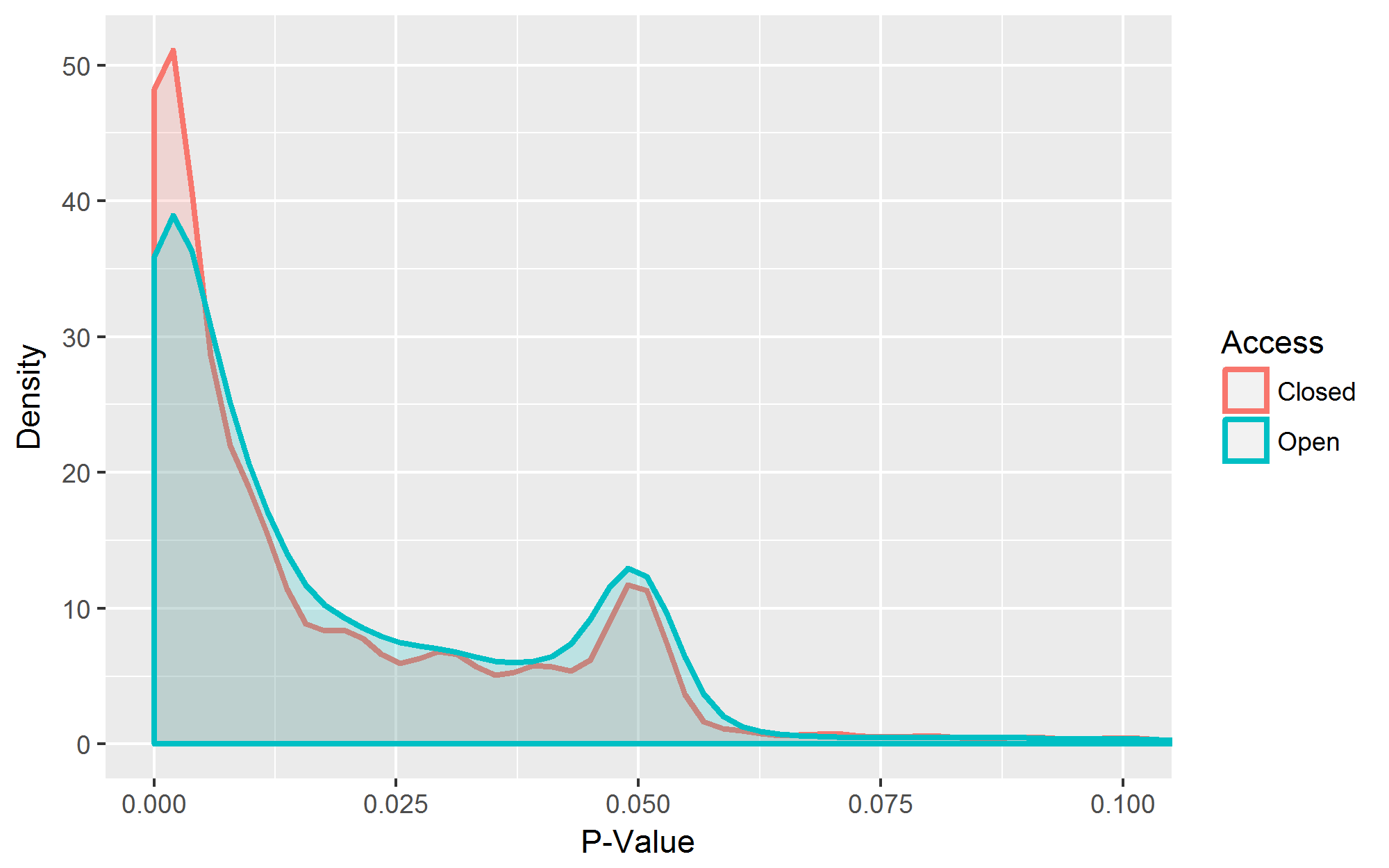


**Figure S2 – Distribution of scraped p-values between 0 and 0.1 by Access Type.** Journals with “Both” access type excluded. Closed access (red) vs. Open Access (blue). The peaks at 0.001 and 0.05 represent large numbers of p-values reported as P < 0.001 or P < 0.05, respectively.

**Distribution of FDR Estimates by Journal Impact Factor**


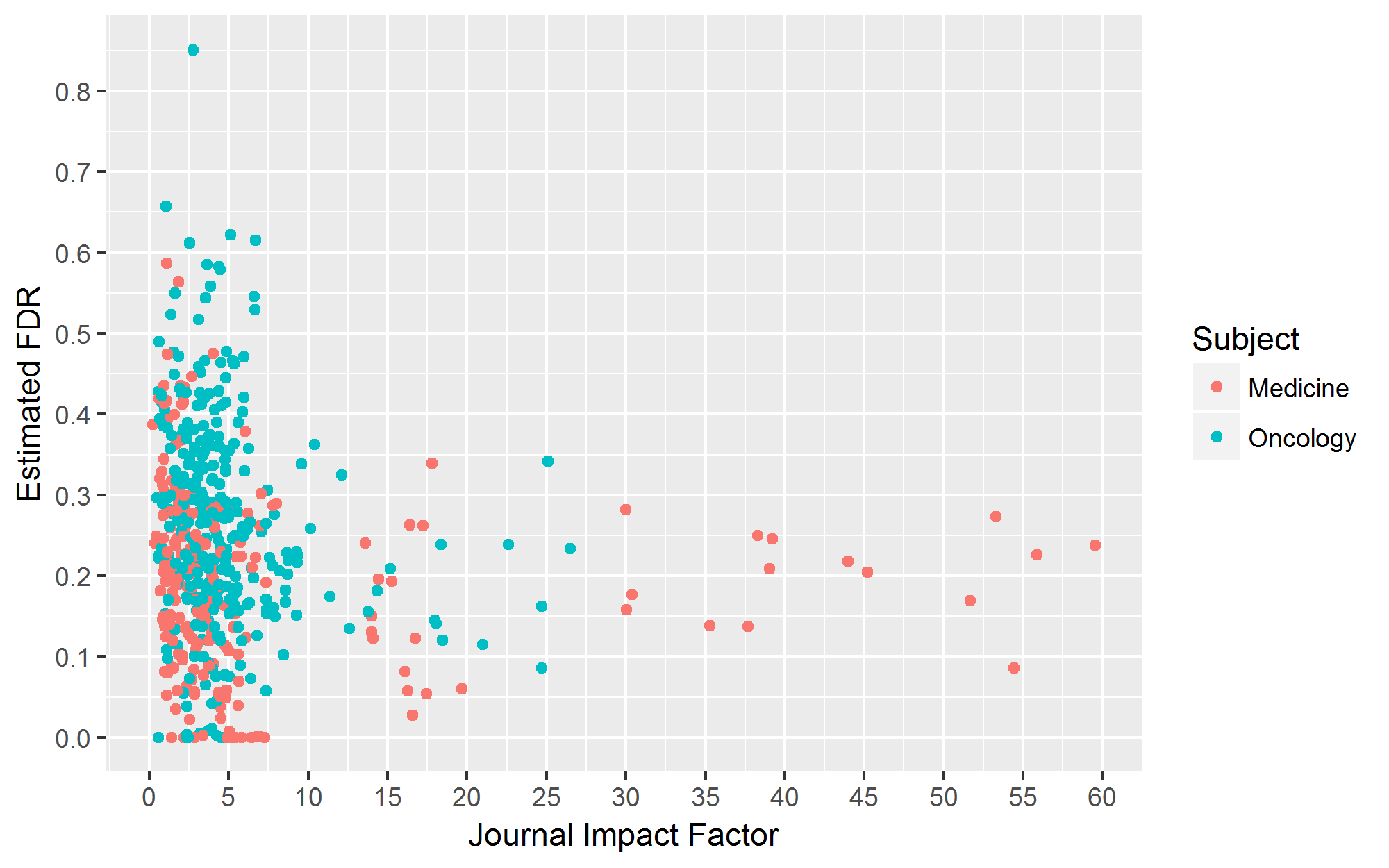


**Figure S3 – Estimated FDR and JIF by Subject.** All journals and all years included. Medicine journals (red) and Oncology journals (blue). JIF for medicine journals ranged from 0.206 to 56.558. JIF for oncology journals ranged from 0.474 to 26.509.


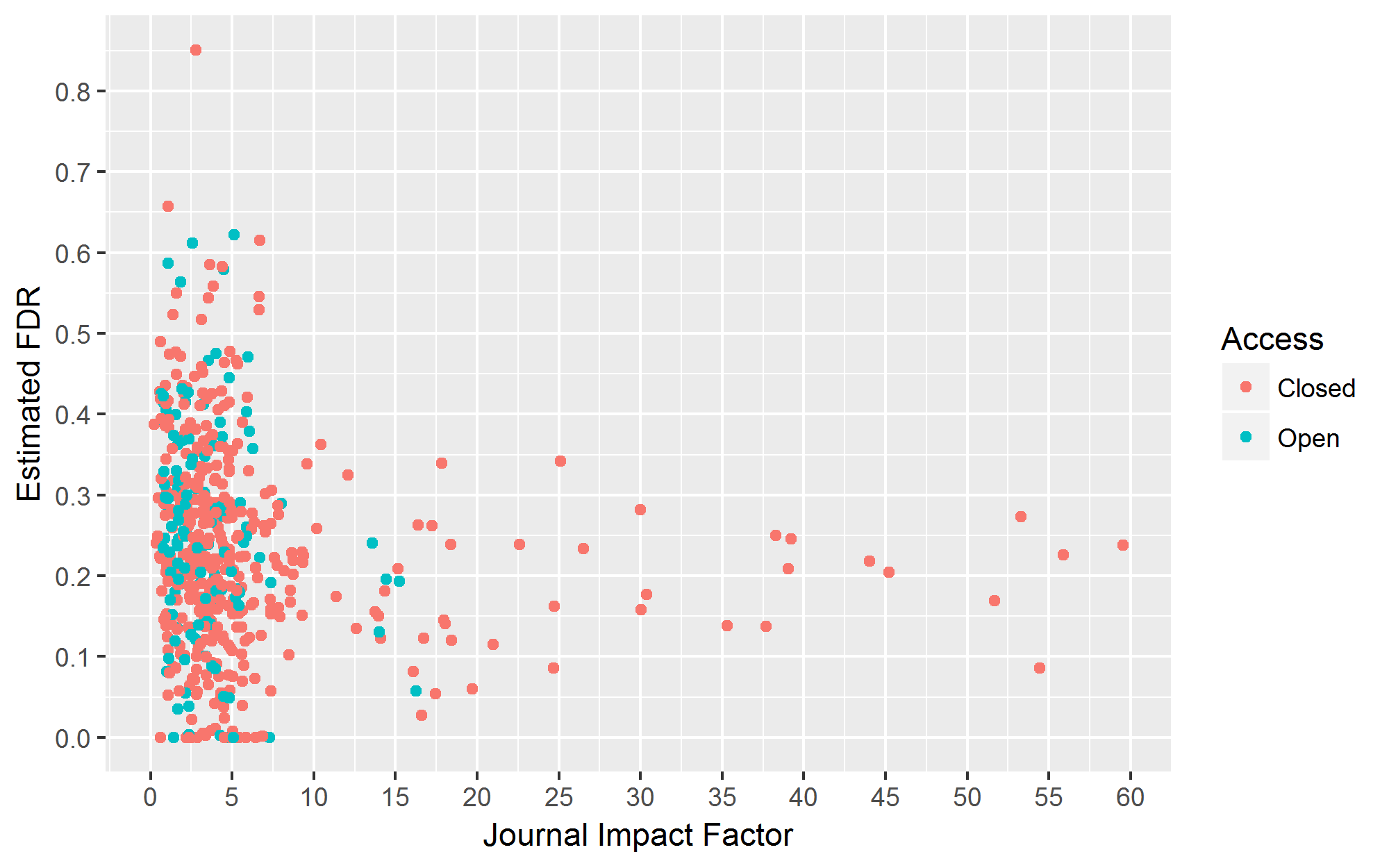


**Figure S4 – Estimated FDR and JIF by Access Type**. Journals with “Both” access type excluded. Closed access (red) vs. Open Access (blue). JIF for closed access journals ranged from 0.206 to 56.558. JIF for Open Access journals ranged from 0.656 to 16.269.


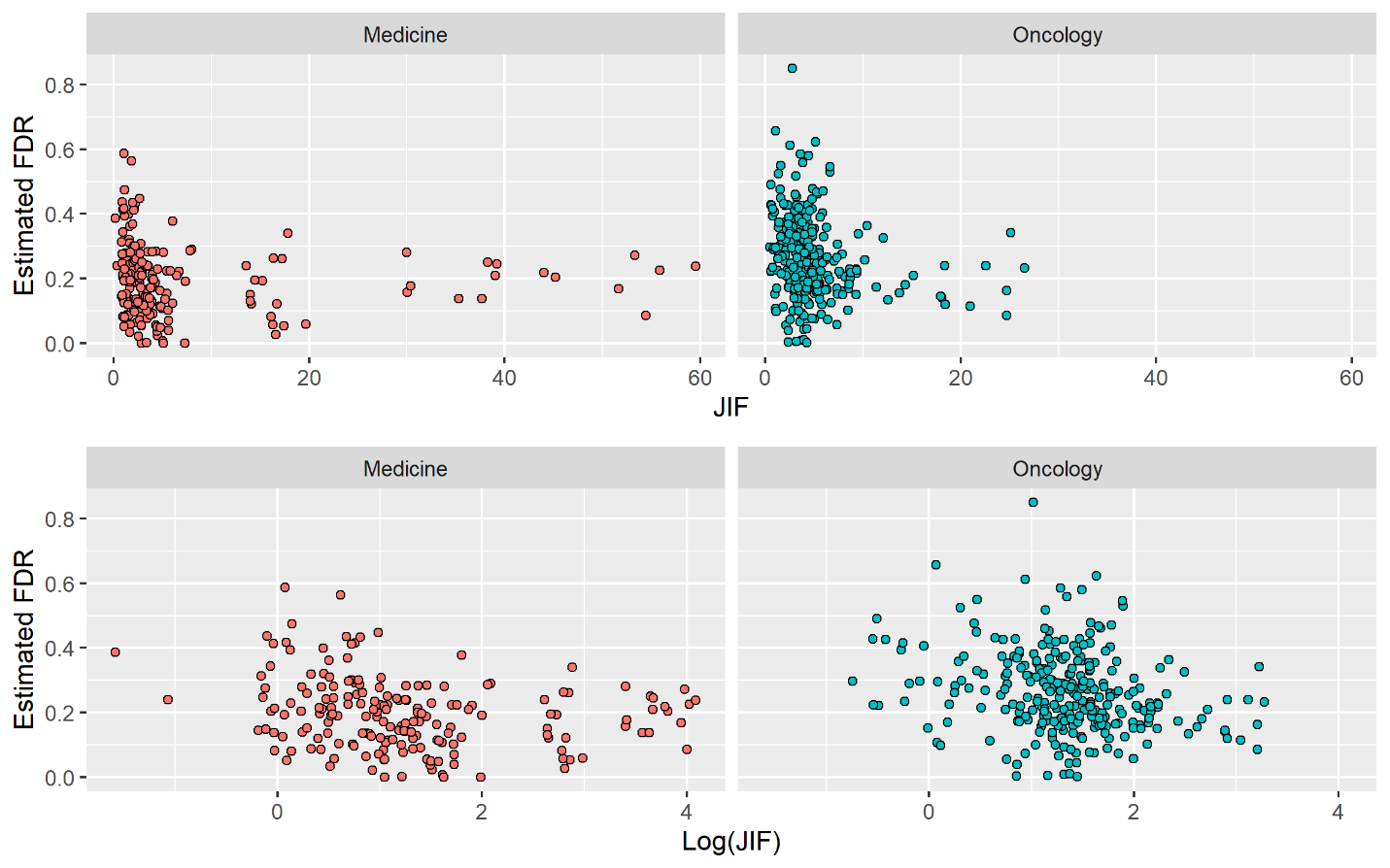


**Figure S5 – Relationship between FDR and JIF by Subject**. A curvilinear relationship appears to exist between JIF and Estimated FDR. The application of a natural logarithm transform of JIF improves the linearity of the relationship.
